# Selenoprotein P Status Correlates to Cancer-Specific Mortality in Renal Cancer Patients

**DOI:** 10.1371/journal.pone.0046644

**Published:** 2012-10-09

**Authors:** Hellmuth A. Meyer, Tobias Endermann, Carsten Stephan, Mette Stoedter, Thomas Behrends, Ingmar Wolff, Klaus Jung, Lutz Schomburg

**Affiliations:** 1 Institute of Physiology, Charité - Universitätsmedizin Berlin, Berlin, Germany; 2 Department of Urology, Charité - Universitätsmedizin Berlin, Berlin, Germany; 3 Institute for Experimental Endocrinology, Charité - Universitätsmedizin Berlin, Berlin, Germany; 4 Berlin Institute for Urologic Research, Berlin, Germany; Johns Hopkins University, United States of America

## Abstract

Selenium (Se) is an essential trace element for selenoprotein biosynthesis. Selenoproteins have been implicated in cancer risk and tumor development. Selenoprotein P (SePP) serves as the major Se transport protein in blood and as reliable biomarker of Se status in marginally supplied individuals. Among the different malignancies, renal cancer is characterized by a high mortality rate. In this study, we aimed to analyze the Se status in renal cell cancer (RCC) patients and whether it correlates to cancer-specific mortality. To this end, serum samples of RCC patients (n = 41) and controls (n = 21) were retrospectively analyzed. Serum Se and SePP concentrations were measured by X-ray fluorescence and an immunoassay, respectively. Clinical and survival data were compared to serum Se and SePP concentrations as markers of Se status by receiver operating characteristic (ROC) curve and Kaplan-Meier and Cox regression analyses. In our patients, higher tumor grade and tumor stage at diagnosis correlated to lower SePP and Se concentrations. Kaplan-Meier analyses indicated that low Se status at diagnosis (SePP<2.4 mg/l, bottom tertile of patient group) was associated with a poor 5-year survival rate of 20% only. We conclude that SePP and Se concentrations are of prognostic value in RCC and may serve as additional diagnostic biomarkers identifying a Se deficit in kidney cancer patients potentially affecting therapy regimen. As poor Se status was indicative of high mortality odds, we speculate that an adjuvant Se supplementation of Se-deficient RCC patients might be beneficial in order to stabilize their selenoprotein expression hopefully prolonging their survival. However, this assumption needs to be rigorously tested in prospective clinical trials.

## Introduction

Selenium (Se) is an essential trace element needed for the biosynthesis of proteins containing the 21^st^ proteinogenic amino acid selenocysteine. Among the functionally characterized enzymatic active selenoproteins are five different glutathione peroxidases (GPx), three iodothyronine deiodinases (DIO), three thioredoxin reductases (TXNRD), and a number of unique enzymes implicated in Se transport, endoplasmic reticulum function, repair of oxidized proteins, Ca signaling, and other catalytic functions [1], [2]. Inherited diseases in human selenoprotein genes are rare and only known from case reports describing a form of congenital muscular dystrophy due to mutations in selenoprotein N (*SELN*) [3]. More complex disease syndromes involving or not growth delay, CNS atrophy and thyroid hormone metabolism defects are described in children with inherited mutations in key genes of selenoprotein biosynthesis (*SBP2* or *SEPSECS*) [4].

Single nucleotide polymorphisms (SNPs) have been described in a number of selenoprotein genes and are associated with the individual response to Se supplementation [5], [6], inflammatory cytokine expression [7] and, most importantly, cancer susceptibility [8]–[11]. In this respect, functionally important cancer-related SNPs have been reported in several selenoprotein genes including *GPx1*, *GPx4*, *TXNRD1* and the circulating Se transport protein selenoprotein P (*SePP*) [8], [9], [12], [13]. These findings provide genetic evidence for a contribution of selenoproteins to cancer risk [11].

This notion is in agreement with the majority of studies comparing Se intake or Se status with tumorigenesis in both experimental animals and clinical analyses [14]. Besides prevention, there is considerable interest in Se for cancer therapy as cancer cells prove especially sensitive to certain selenocompounds [15]–[17]. However, successful clinical studies in this direction have not yet been conducted. Many case control studies have indicated that Se concentrations in blood are lower in cancer patients compared to healthy controls, as reported in e.g. bladder cancer [18], hepatocellular carcinoma [19], colorectal adenoma [20] or prostate cancer [21]. Only recently, respective analyses have been complemented by measurements of SePP, as it is the major selenoprotein in human blood [22] representing a reliable biomarker for Se status [23] or Se supplementation trials [24]. Collectively, the current data support the hypothesis that low Se status increases cancer risk, and that a malignant disease decreases the Se status in the patient even further thereby closing an unfavorable feedforward cycle.

We have recently compared serum Se and SePP concentrations in prostate cancer patients, verifying that both parameters correlate reliably in Se-deficient individuals and may improve prostate cancer diagnosis [25]. Now, we took advantage of a serum bank of renal cell carcinoma (RCC) patients collected at the time of cancer diagnosis and stored deep frozen over more than 60 months. A comparison to clinical and survival data indicated that Se and SePP concentrations were reduced in RCC patients compared to controls. Moreover, low Se and SePP concentrations in RCC patients were associated with cancer severity, i.e., cancer grade and stage. Importantly, mortality rate was inversely associated with SePP concentrations at diagnosis, thus raising the issue of whether an adjuvant Se supplementation supporting the usual therapeutic measures may improve survival outcome of Se-deficient RCC patients.

## Materials and Methods

### Patients and Samples

In total, serum samples from 62 patients from the Department of Urology, University Hospital Charité, were analyzed. The analyses were approved by the medical ethics committee of the Charité hospital in Berlin, Germany. Written informed consent from all participants involved in the study was obtained prior to analysis. The selection criterion for the inclusion of patients into our retrospective analysis was the availability of comprehensive follow-up information and suitable sample material (i.e. unthawed aliquots of at least 0.5 ml serum per patient). Blood samples had been taken before any diagnostic or therapeutic procedure. After sample collection, the sera had been stored in aliquots at −80°C and were analyzed retrospectively. Sample size determinations and power calculations were based on our previous study results on SePP concentrations in prostate cancer [25] assuming a two-sided alpha error of 5% and a power of 80% for changes of 1 SD between control and RCC subjects. Under these assumptions, a sample size of 32 subjects (16 controls and 16 patients) was calculated as the minimum needed for our study. In the end, we analyzed bigger groups as there were more samples available that qualified for our analysis according to our inclusion criteria. The serum samples were stratified in two groups: samples from 41 patients receiving radical nephrectomy for renal cell carcinoma (RCC) (median age, 63 y; range, 48–83 y; ratio of females, 32%; collected between 2003 and 2005) and control samples from 21 healthy persons showing “no evidence of malignancy” (NEM) (median age, 51 y; range, 29–75 y; ratio of females, 33%; collected between 2008 and 2009). The pathological staging and grading were pT1 (n = 15), pT2 (n = 1), pT3 (n = 22), pT4 (n = 3) and G1 (n = 5), G2 (n = 24), G3 (n = 9), G4 (n = 3).

### Methods

SePP and Se analyses were conducted in a remote lab from the Department of Urology in a blinded fashion with respect to patient identity and characteristics. SePP concentrations were determined from serum samples by an immunoluminometric sandwich assay as described [26]. Total Se concentrations were determined by X-ray fluorescence, using a benchtop total reflection X-ray fluorescence (TXRF) spectrometer (Picofox™ S2, Bruker, Karlsruhe, Germany) as described [27]. Briefly, a Gallium standard was added as internal control to the serum samples, and aliquots were applied to polished quartz glass carriers and dried at room temperature. Se measurements were controlled with a commercial standard serum (Seronorm, Billingstad, Norway) and an atomic absorption standard (1000 mg/ml, Sigma, Taufkirchen, Germany). Mean Se concentrations for the human standard serum (168.7±8.8 µg/l) were in accordance with the corrected values as published in literature [28].

### Statistical Analysis

Statistical calculations were done with SPSS, version 19.0 (SPSS Software, IBM, Munich, Germany) and MedCalc for Windows, version 12.2.1.0 (MedCalc Software, Mariakerke, Belgium). Mann-Whitney U test for unpaired samples was used to evaluate the differences between groups. Pearson P coefficient was used to assess the statistical significance of the correlation between SePP and Se. The correlation coefficient according to Spearman was used to assess the statistical significance between clinicopathological parameters.

The diagnostic accuracy of the markers was evaluated using receiver operating characteristic (ROC) curve analysis with calculations of the area under receiver operating characteristic curve (AUC). Univariate survival analysis was performed according to Kaplan-Meier, and differences in survival curves were assessed with the log rank test. Multivariate survival analysis was performed using the Cox regression model. P<0.05 was considered to indicate statistical significance.

## Results

### Serum Baseline Characteristics of Se and SePP in Cases and Controls

**Figure 1:**
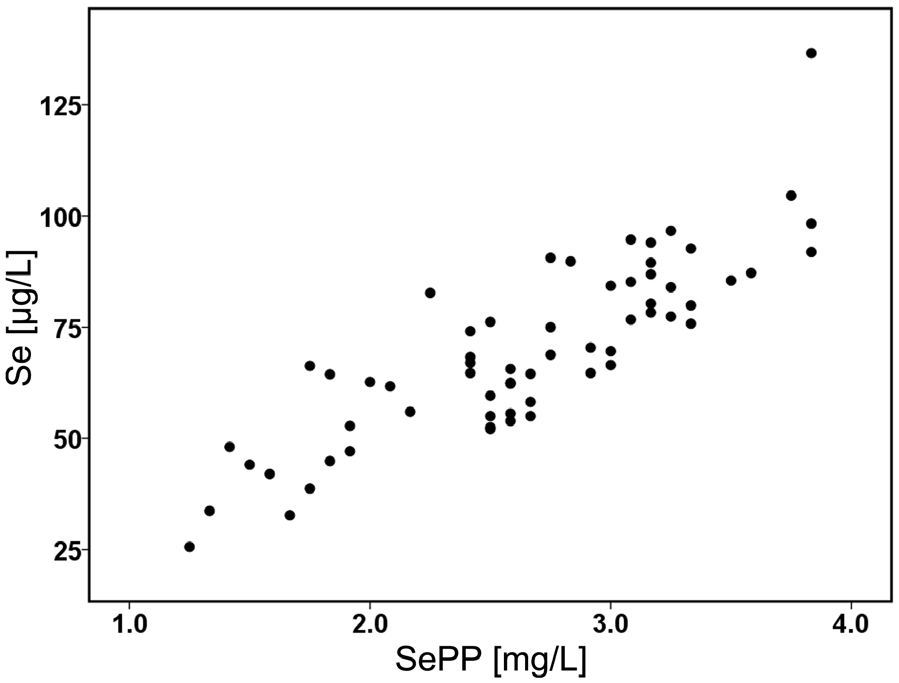
Dotplot analysis of Se and SePP concentrations. Serum Se concentrations were determined by total reflection X-ray fluorescence and serum SePP concentrations were determined by an immunoluminometric sandwich assay. Both markers of Se status correlate linearly as known from Se-deficient individuals (Pearson’s correlation coefficient = 0.849; p<0.001).

In total, 20 out of the 41 patients were diagnosed with metastatic RCC (Table 1). Total Se and SePP concentrations were analyzed in all serum samples as described [27]. The median concentrations of Se and SePP were significantly (*P*<0.001) lower in RCC patients compared to the control group (Table 2). Se and SePP concentrations correlated significantly (*r*_s_ = 0.85; *P*<0.001), as known from populations with marginal Se supply and in contrast to well-supplied individuals [23], [29], [30]. Notably, serum SePP concentrations are known to become saturated on a maximum level once the Se intake and Se status are replete highlighting the suitability of serum SePP as a most useful biomarker for Se status assessment in subjects with an existing or developing Se deficit [31]. The results indicate that our groups consisted of poorly supplied European individuals in which both markers were suitable to reflect the Se status (Figure 1). No significant changes in serum concentration of other mineral nutrients such as iron, zinc or copper were observed between control and RCC patients (data not shown).

**Table 1:** Clinicopathologic characteristics of patients and controls.

Patient characteristics		
Age median (min-max) [y]		62.0 (29–83)
Sex n (%)	Male	42 (67.7%)
	Female	20 (32.3%)
Follow-up time median (min-max) [mo]		39 (0–65)
Survival (only RCC patients, n = 41)	Alive	21 (51.1%)
	Dead	20 (48.9%)
**Tumor characteristics** (RCC patients n = 41)		
Histologic classification n (%)	Clear cell RCC	36 (87.8%)
	Papillary RCC	5 (12.2%)
Tumor size median (min-max) [mm]		55 (20–180)
Pathologic stage n (%)	pT1	15 (36.6%)
	pT2	1(2.4%)
	pT3	22 (53.7%)
	pT4	3 (7.3%)
Grading n (%)	G1	5 (12.2%)
	G2	24 (58.5%)
	G3	9 (22.0%)
	G4	3 (7.3%)
Metastases n (%)	M0	21 (51.1%)
	M1	20 (48.9%)

**Table 2:** Serum concentrations of Se and SePP in RCC patients* and controls.

	NEM (n = 21)		RCC (n = 41)		*P* #
	median (min-max)	lower/upper quartiles	median (min-max)	lower/upper quartiles	
SePP (mg/L)	3.17 (1.8–3.8)	2.7/3.3	2.58 (1.2–3.8)	1.9/2.8	<0.001
total Se (µg/L)	84.3 (44.9–104.6)	72.2/93.4	64.4 (25.6–136.6)	54.5/74.6	<0.001

* Patients were classified by diagnosis; the serum data are presented as median, range, and percentile.

# Mann-Whitney U-Test.

### Serum SePP Concentrations in Relation to Tumor Characteristics

**Figure 2:**
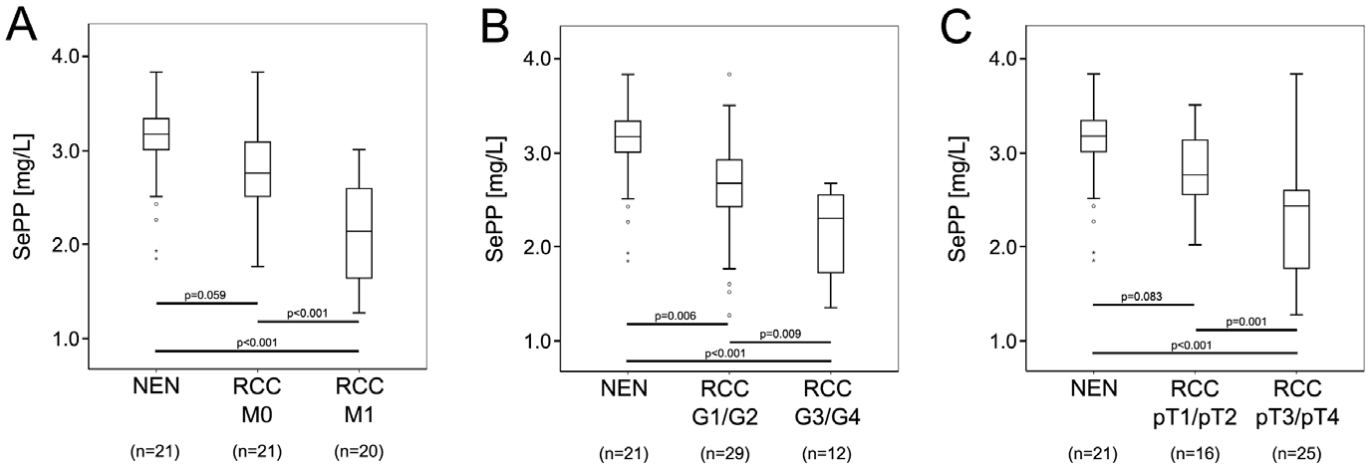
Box Plot analysis of serum SePP concentration in RCC patients and controls. RCC patients were stratified using pathologic tumor characteristics **A**) non metastatic versus metastatic **B**) G1/G2 versus G3/G4 and **C**) pT_1_/pT_2_ versus pT_3_/pT_4._ The corresponding significance levels between groups are given in the graph.

Our cohort showed a significant (*P* = 0.002) difference in age between healthy donors (median age: 51 a (min. 29 a, max. 75 a)) and RCC patients (median age: 63 a (min. 48 a, max. 83 a)). In our previous analyses, a tendency of increasing SePP concentrations with age was observed in healthy adult Danes [32] and Germans [26]. When analyzing our groups of NEM and RCC subjects separately, no significant correlation of SePP concentrations with age was observed. Next, we analyzed the RCC patients without control individuals stratified by their pathologic tumor characteristics. We found that lower serum concentrations of SePP were significantly associated with more aggressive cases of RCC, as indicated in Figure 2. The tested subgroups were: non metastatic versus metastatic (median SePP: 2.8 to 2.1 mg/l; *P*<0.001); pT_1_/pT_2_ versus pT_3_/pT_4_ (median SePP: 2.8 to 2.4 mg/l; *P* = 0.009) and G1/G2 versus G3/G4 (median SePP: 2.7 to 2.3 mg/l; *P* = 0.001). No significant differences between the tested groups concerning age were found.

### Diagnostic and Prognostic Potential of Serum SePP Expression

**Figure 3:**
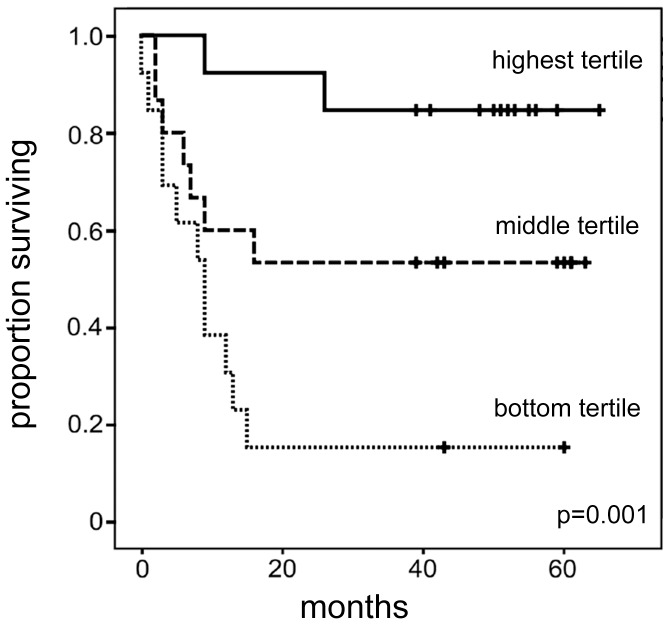
Kaplan-Meier estimates of cancer-specific survival in RCC patients according to SePP serum concentrations. RCC patients (n = 41) were categorized into relatively high, moderate or low Se status by calculating tertiles of serum SePP as a reliable circulating biomarker (highest tertile: [SePP] >2.7 mg/l; middle tertile: [SePP] = 2.4–2.7 mg/l; bottom tertile: [SePP] <2.4 mg/l). Low Se status as reflected by serum SePP concentrations in the bottom tertile at time of diagnosis is associated with poor survival odds.

To determine the diagnostic potential of serum SePP between control and RCC cases, ROC curve analysis was performed, reaching an AUC of 0.77 (95%Cl, 0.64–0.91). Cumulative survival curves were calculated according to Kaplan-Meier. The conventional prognostic parameters such as tumor grade and pT status reached significance for survival in our cohort (data not shown). Overall, low SePP serum concentrations (<2.4 mg/l, representing the bottom tertile of the RCC patients) were found in patients with higher tumor stage, grade and with metastases; low SePP concentrations were likewise significantly associated with shorter survival of RCC patients (Figure 3). In multivariate Cox-analysis (including SePP, metastatic status, tumor grade and pT status) serum concentration of SePP reached not the status of an independent prognostic factor.

## Discussion

The interaction of Se and cancer is a vividly disputed issue, and especially the question of whether Se supplementation may serve chemopreventive purposes is controversially discussed [33]. Part of the conflict appears to base on the misconception about the importance of baseline Se status. Our results presented here are derived from healthy probands and RCC patients residing in a Se poor area, i.e. central Europe. Respective analyses with RCC patients from better supplied areas might not necessarily yield the same results, as individuals with higher baseline Se status have a higher Se body store and reserve which may become mobilized in times of need [34]. Our findings in RCC verify the notion that cancer patients residing in Se poor areas have a relative Se deficiency compared to healthy controls, and that it widens with cancer stage and grade. It is likely that both a Se deficiency-dependent predisposition and a cancer-related progressive Se decline contribute to this finding. These tendencies are not specific for RCC and have also been described in a number of other malignancies [9], [18]–[21]. It has been estimated that the cut-off for Se sufficiency ensuring maximal expression of SePP as the most demanding circulating selenoprotein is reached at plasma Se concentrations of 90–124 µg/l [23], [24]. This notion is in agreement with our latest analysis in well-supplied US Americans, where the average Se concentration was 142 µg/l and SePP concentrations had thus reached a maximum and were independent of serum Se [6]. Most of our patients and controls had been below this level already at diagnosis indicating that they were at risk of developing a clinically relevant Se deficiency during the course of the disease.

Average Se intake and blood Se concentrations differ markedly between the countries. Changes in food quality in combination with changing supplementation or dietary habits affect the personal Se status [27], [35]. Some countries have experienced a population-wide alteration in Se intake during the last decades, e.g. the UK, Finland and the U.S.A. In the UK, import of Se-rich US American wheat has declined and been replaced by locally produced items causing a generally lower Se intake and Se status [36]. In contrast, Finland developed a systematic agronomic biofortification program which increased Se contents in plant and animal foodstuff causing a generally improved daily Se intake [37]. In the U.S.A., it seems as if Se supplementation of some dietary products (health food items) and other personal measures (high frequency of taking multimineral supplements) have increased average Se intake during the last decades.

Especially this trend of uncontrolled micronutrient (trace elements and vitamins) supplementation bears the risk of over-supplementation [38]. More importantly for cancer research, it may have precluded a successful replication of the NPC trial which demonstrated a high chemopreventive activity of supplementary Se intake on lung, colorectal, and prostate cancer risk [39]. The respective follow-up study trying to replicate the prostate cancer chemopreventive effects enrolled probands with significantly higher baseline Se status, and yielded no positive supplementation effects [40]. These two huge and well-controlled Se supplementation trials [39], [40] dominate respective meta-analyses. As they have been conducted in the U.S.A. where Se intake is sufficient [35], their uncritical perception caused biased conclusions and premature extrapolations [41] when uncritically applied to marginally supplied populations.

Our data highlight again the importance of health for Se status, i.e., the interaction of disease severity and Se-deficiency. Only very few patients with relatively low Se status characterized by SePP concentrations below 2.4 mg/l at time of diagnosis survived our 5-year observation period. A similar trend of low Se status correlating to fundamentally higher mortality rates has been noted in European studies with sepsis patients [42], [43]. A respective Se supplementation trial improved 28 day survival in severe sepsis [43]. Autoimmune thyroid disease is another field where Se is used as an adjuvant treatment option. Several respective supplementation trials have reported on successfully reducing autoantibody load in Hashimoto thyroiditis by Se supplementation [44]. Notably, these positive studies have again been reported solely from European countries involving patients with marginal Se status [45].

The most important question thus relates to the clinical meaning of our analysis, i.e., whether Se-deficient RCC patients should be supplemented with Se or not. This aspect can only be resolved by conducting a prospective clinical supplementation trial. Our results support such an approach in patients who have a documented Se deficit, since well-supplied patients may not profit from additional Se.

The situation is different in better supplied areas, e.g. the U.S.A., where average baseline Se concentrations have been determined as 122–152 µg/l [6], [46]. However, from our case-control data, it can not be decided whether the RCC patients had a low Se status because of predisposition or secondary to malignancy, but the correlations observed between severity and Se deficit argue for a negative influence of the tumor(s) on the Se status. This interaction may relate to an increased tone of cancer-related proinflammatory cytokines known to impair Se metabolism [47], [48].

Importantly, our study gives hints towards the medical meaning of a severely decreased Se status in terms of poor survival odds. This has, to the best of our knowledge, not been described previously for RCC. It is known from chronic kidney disease that serum Se and extracellular GPx3 activities are declining in proportion to disease severity [49]. Such a Se deficit may deprive Se-sensitive tissues including the endocrine glands, the CNS and the kidneys of the essential trace element needed for the biosynthesis of protective selenoenzymes. Increased damage may result aggravating the disease and reducing survival odds.

The kidney plays a central role in Se homeostasis by excreting selenosugars (1-beta-methylseleno-N-acetyl-d-galactosamine) as normal end products of Se metabolism [50]. Upon excessive Se intake, also trimethylselenonium is formed and disposed into the urine. SePP is normally not secreted however it passes into the primary glomerular filtrate from where it becomes re-absorbed by proximal tubule epithelial cells via a receptor-mediated mechanism [51], [52]. It remains to be studied in how far gastrointestinal uptake, hepatic organification or renal re-absorption is impaired in RCC patients and contribute to the observed Se deficit. Such mechanistic studies may provide the missing impetus and rationale needed for conducting a respective supplementation study in Se-deficient RCC patients.

Our study has some limitations. Despite the significant interaction of survival odds and SePP concentrations at time of diagnosis, the number of patients analyzed in the present study is relatively small. However, the group sizes actually investigated were consistent with type I and type II error-specific preconditions (alpha = 5%; beta = 80%) in the study design calculations. In addition, the median follow-up time is comparably long and the interactions appear strong providing a new and important insight into the importance of Se status for survival in RCC patients. In addition, the pathological pathways responsible for the effects observed are largely unknown at present. More detailed mechanistic studies are needed in order to characterize alterations in Se metabolism and SePP biosynthesis under pathological conditions with an emphasis on kidney as a central organ for Se status control.
